# Distal Femur Intraarticular Fracture in a Late Arthritic Knee Treated With Osteosynthesis and Computer Navigation Assisted Primary Total Knee Replacement: A Case Report

**DOI:** 10.7759/cureus.29102

**Published:** 2022-09-13

**Authors:** Sujoy K Bhattacharjee, Abhishek Mehta

**Affiliations:** 1 Orthopaedics, Sarvodaya Hospital & Research Centre, Faridabad, IND; 2 Orthopaedics, Nehru Shatabdi Chikitsalaya, Singrauli, IND

**Keywords:** computer assisted surgery, primary tka, osteosynthesis, distal femur intraarticular medial condyle, stress fracture

## Abstract

The incidence of osteoporosis and osteoarthritis is on the rise. What further complicates the scenario is a stress fracture in a weight-bearing joint such as a knee in the presence of arthritis, making the treatment challenging. Prolonged immobilization associated with osteosynthesis increases morbidity and mortality in elderly patients. Primary total knee arthroplasty (TKA) has been advocated as a treatment modality in patients with distal femoral fractures who already have painful arthritic knees. Most of these injuries get treated using a hinged prosthesis. However, there are concerns about the high rate of loosening and mechanical failure of this type of prosthesis. This report presents a distal femur intraarticular fracture nonunion in the late arthritic knee, which is a rare presentation as proximal tibia stress fractures are more common. This was treated with osteosynthesis, and computer navigation assisted primary total knee replacement using medial pivot knee in a 54-year-old male with a body mass index of 38. Based on clinical and radiographic evidence, primary total knee replacement and plate osteosynthesis are viable options for distal femur fractures with osteoarthritis using computer navigation. While limiting the number of procedures, it meets two prerequisites: early weight bearing, limiting decubitus-related complications, and early mobilization leading to patient autonomy.

## Introduction

Distal femur fractures in the elderly can occur even after low-energy trauma in patients with knee osteoarthritis. Pre-existing osteoporosis makes the treatment difficult compared to the younger population. Internal fixation is usually tricky due to osteoporosis and metaphyseal comminution. In such patients co-existing osteoarthritis of the knee complicates the issue further as the knee joint remains painful with impaired knee function even after the union. Prolonged immobilization associated with osteosynthesis increases morbidity as well as mortality in elderly patients [1,2].

Stress fractures are overuse injuries of the bone. Stress fractures in the elderly are mainly due to osteoporosis [3], post-traumatic deformity [4], deformed degenerated knees [5-10], and post-knee arthroplasty [11-14].

Treatment of stress fracture secondary to osteoarthritis is especially challenging because malalignment secondary to arthritic deformity leads to undue stress at the fracture site, which prevents union, fixation failure, rapid progression of arthritis, and stiffness [15,16]. Concomitant osteoporosis makes the management even more difficult. The issue in treating such a patient is difficult or even impossible to achieve a stable bone reconstruction using osteosynthesis due to osteoporosis and the necessity for early functional recovery.

Surgical options for these patients are Osteosynthesis in the first stage, followed by total knee arthroplasty in 2nd stage; Total knee replacement with stem extensions, and osteosynthesis in a single stage [17].

## Case presentation

A 53-year-old male presented in the outpatient department of Sarvodaya Hospital Faridabad Haryana with complaints of pain and deformity in both knees for two months with more pain on the right side. The patient was also unable to walk for 2-3 years and was confined to the bed. There was no history of trauma. The pain was spontaneous onset with a gradual increase in severity and gradually progressing deformity, with details of deformity in Table 1.

**Table 1:** Details of deformity in both the knees

Knee	Varus Deformity	Flexion Deformity
Right	15 degrees	20 degrees
Left	20 degrees	20 degrees

**Figure 1:**
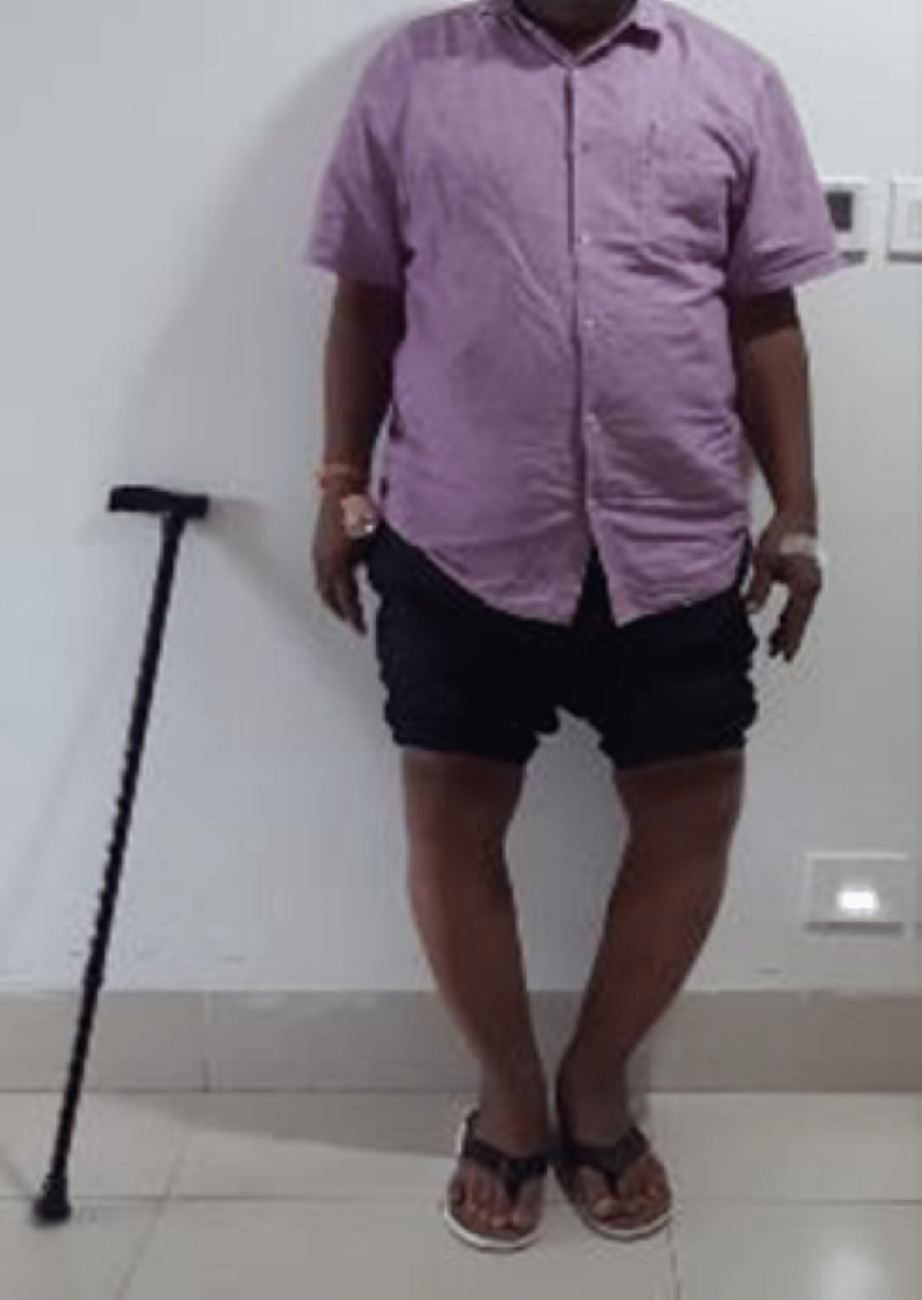
Pre-operative photograph showing varus deformity

**Figure 2:**
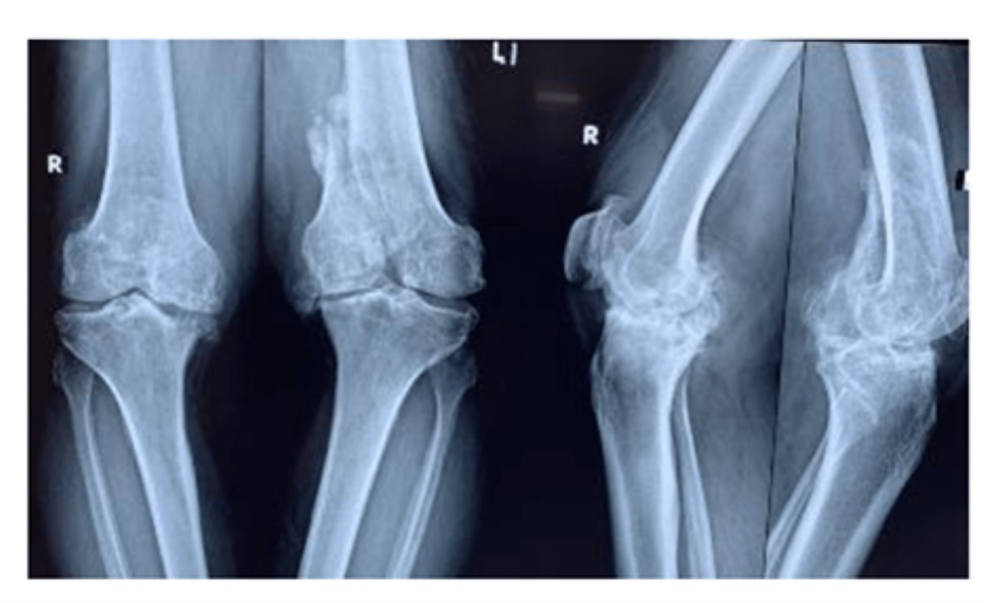
Showing severe Osteoarthritis of both knees

**Figure 3:**
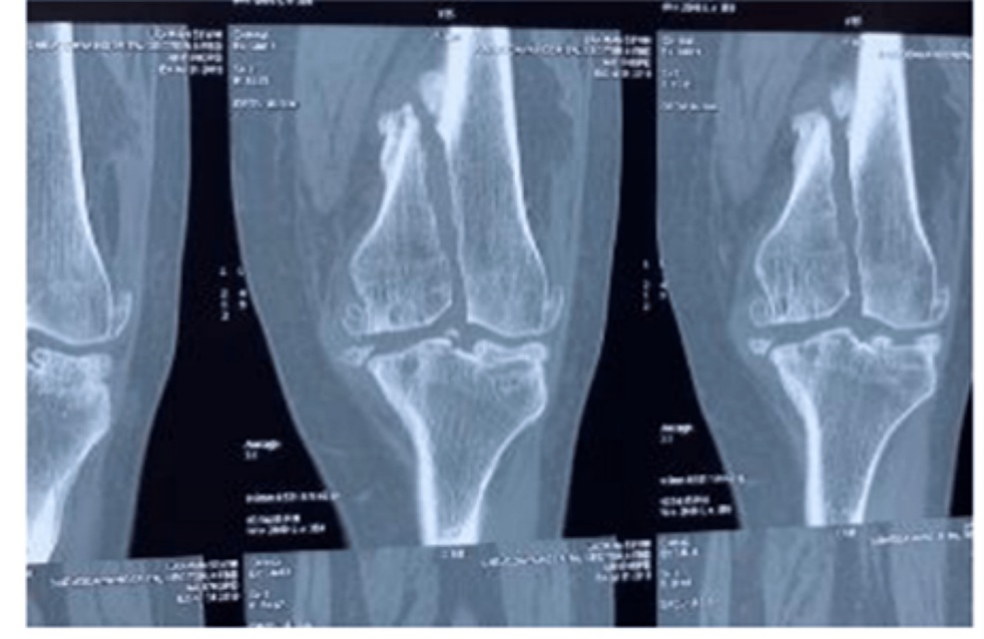
CT Scan (coronal cuts) images of the left knee showing distal femur stress fracture with intra-articular extension.

On examination, the patient had varus deformities of 15 degrees and 20 degrees on the right and left, respectively, and flexion deformities of 20 degrees on both sides (Table 1, Fig 1). Weight-bearing x-rays of both knees (Fig 2), full-length x-rays of both, and a CT scan left knee were ordered (Fig 3). On x-rays, there was severe osteoarthritis (KL scale of 5) of both knees along with intraarticular stress fracture of the medial femoral condyle of the left knee.

**Figure 4:**
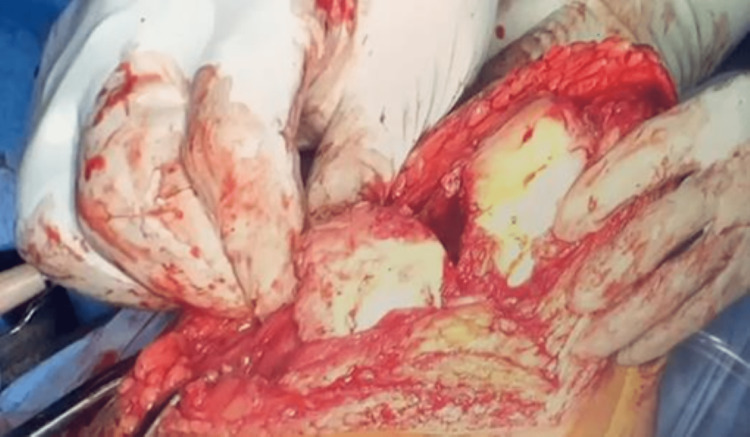
Intraoperative photograph showing the intra articular medial femoral condyle fracture

**Figure 5:**
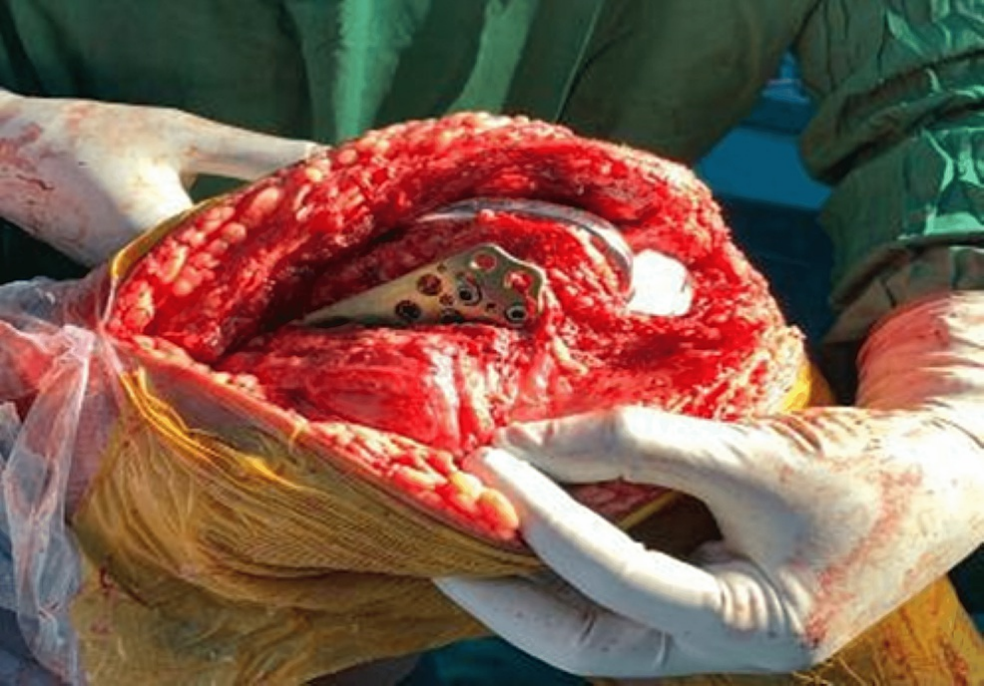
Intraoperative photograph showing the knee replacement implant as well as the locking plate used to fix the fracture

**Figure 6:**
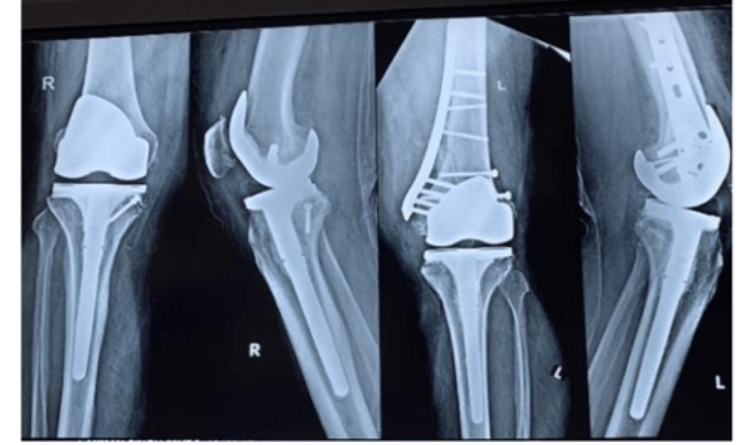
Immediate Post-operative X rays of both knees (AP and Lateral Views)

The patient was advised for a total knee replacement of both knees and osteosynthesis of the left distal femur. After a pre-anesthetic check-up and obtaining informed consent patient was taken up for surgery. A total knee replacement on the right side was done with a medial pivot knee with tibia stem extension. On the left side, osteosynthesis of the medial femur condyle was done using the Right distal femur plate, followed by total knee replacement using primary femur prosthesis and tibia prosthesis with stem extension (medial pivot knee) (Fig 4-6).

**Figure 7:**
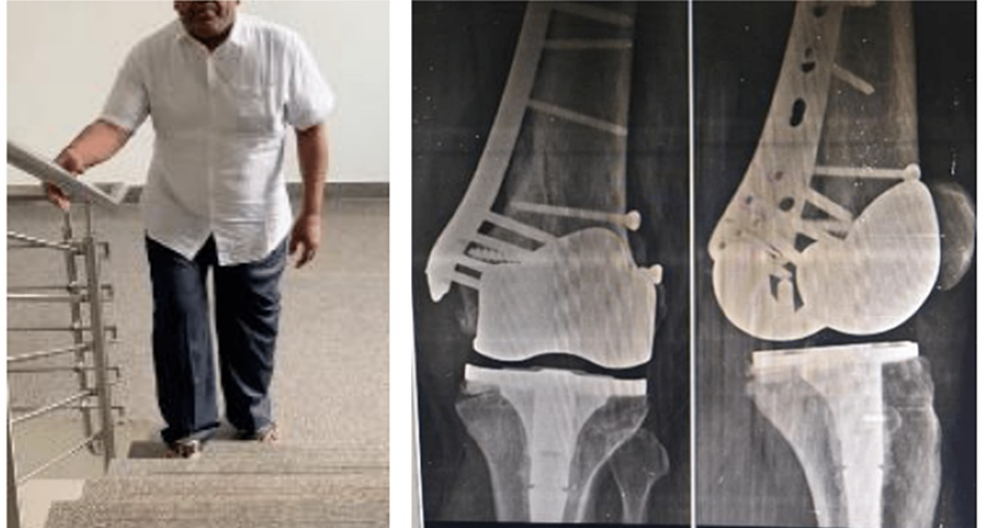
Nine months follow-up showing well-united fracture and a satisfied patient able to carry out daily activities independently.

**Figure 8:**
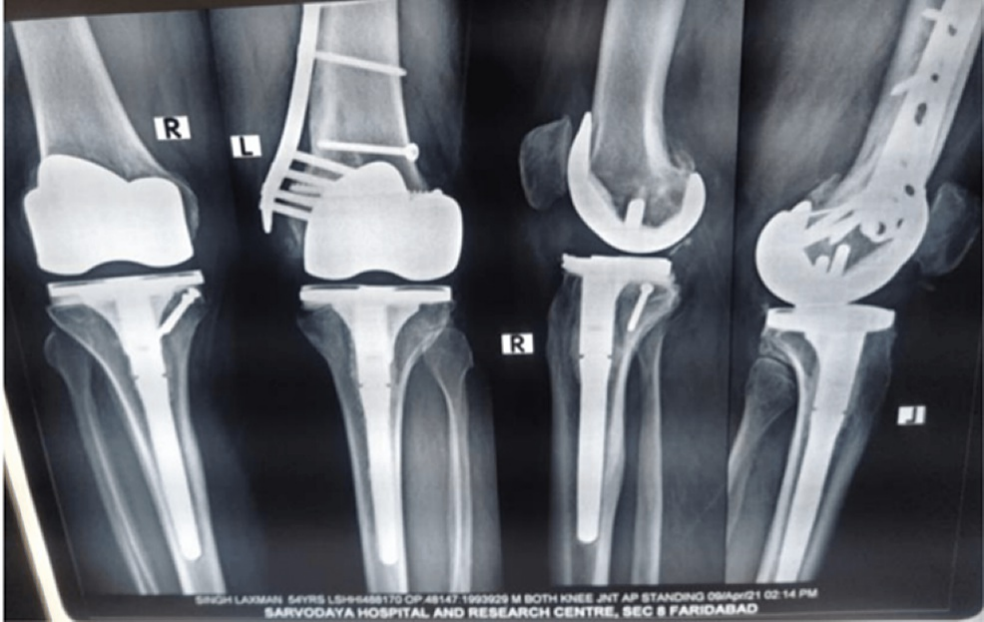
20-month follow-up X-rays showing the complete union of the fracture.

The patient was started with knee mobilization on the same day of surgery and Partial weight bearing the next day. The patient was followed up for three years showing excellent bony union with a plate in situ and a well-integrated Knee prosthesis with a range of motion at knee 0-140 (Fig 7, 8).

## Discussion

Distal femur stress fractures are less common than proximal tibia fractures in the arthritic knee. Malalignment due to osteoarthritis can increase the stress at the fracture site, leading to a nonunion or delayed union. Early achievement of painless movements and ambulation is desirable. Conservative treatment using casts or braces is associated with prolonged immobilization and can result in increased knee stiffness even after aggressive physical therapy after immobilization.

Current methods employing two-stage surgery cause significant hampering of mobility of patients as well as the persistence of pain due to osteoarthritis. In single-stage surgery, the technique of femur stem extension does not provide the desirable stability. Our technique used the principle of rigid anatomical fixation along with knee replacement to achieve early weight bearing and knee mobility [18].

The main highlights were computer navigation and medial pivoting knee implant [19,20]. The conventional method of taking cuts using an intramedullary guide cannot be employed after fixation with a locking compression plate; therefore, computer navigation was used. A cruciate retaining knee could not be used in this case, and the posterior cruciate ligament had to be sacrificed as the patient had severe varus and flexion deformity. A conventional posterior sacrificing knee implant’s cam and post mechanism would have obstructed distal femur screws. So, a medial pivot knee was used, which allowed us to optimally balance the knee without taking a femoral box cut and hence removing the problem of the cam and post obstructing the plate screws.

## Conclusions

Based on clinical and radiographic findings, primary total knee replacement and plate osteosynthesis are viable options for distal femur fractures with osteoarthritis.

## References

[1] Bell KM, Johnstone AJ, Court-Brown CM, Hughes SP (1992). Primary knee arthroplasty for distal femoral fractures in elderly patients. J Bone Joint Surg Br.

[2] Marks DS, Isbister ES, Porter KM (1994). Zickel supracondylar nailing for supracondylar femoral fractures in elderly or infirm patients: a review of 33 cases. J Bone Joint Surg Br.

[3] Devas M (1975). Stress fractures.

[4] Thomas M, Schofield CB, Unwin AJ (1991). Tibial plateau fractures followed by stress fractures. J Bone Joint Surg Br.

[5] Reynolds MT (1972). Stress fractures of the tibia in the elderly associated with knee deformity. Proc R Soc Med.

[6] Wheeldon FT (1961). Spontaneous fractures in the shin in the presence of knee deformities. Proc R Soc Med.

[7] Young A, Kinsella P, Boland P (1981). Stress fractures of the lower limb in patients with rheumatoid arthritis. J Bone Joint Surg Br.

[8] Satku K, Kumar VP, Pho RW (1987). Stress fractures of the tibia in osteoarthritis of the knee. J Bone Joint Surg Br.

[9] Martin LM, Bourne RB, Rorabeck CH (1988). Stress fractures associated with osteoarthritis of the knee. a report of three cases. J Bone Joint Surg Am.

[10] Learmonth ID, Grobler G (1990). Sequential stress fractures of the tibia associated with osteoarthritis of the knee. a case report. S Afr J Surg.

[11] Rand JA, Coventry MB (1980). Stress fractures after total knee arthroplasty. J Bone Joint Surg Am.

[12] Manzotti A, Confalonieri N, Pullen C (2008). Intra-operative tibial fracture during computer assisted total knee replacement: a case report. Knee Surg Sports Traumatol Arthrosc.

[13] Seon JK, Song EK, Yoon TR, Seo HY, Cho SG (2007). Tibial plateau stress fracture after unicondylar knee arthroplasty using a navigation system: two case reports. Knee Surg Sports Traumatol Arthrosc.

[14] Hoke D, Jafari SM, Orozco F, Ong A (2011). Tibial shaft stress fractures resulting from placement of navigation tracker pins. J Arthroplasty.

[15] Thomson AB, Driver R, Kregor PJ, Obremskey WT (2008). Long-term functional outcomes after intra-articular distal femur fractures: ORIF versus retrograde intramedullary nailing. Orthopedics.

[16] Vallier HA, Hennessey TA, Sontich JK, Patterson BM (2006). Failure of LCP condylar plate fixation in the distal part of the femur. a report of six cases. J Bone Joint Surg Am.

[17] Sourlas I, Papachristou G, Pilichou A, Giannoudis PV, Efstathopoulos N, Nikolaou SA (2009). Proximal tibial stress fractures associated with primary degenerative knee osteoarthritis. Am J Orthop.

[18] Mittal A, Bhosale PB, Suryawanshi AV, Purohit S (2013). One-stage long-stem total knee arthroplasty for arthritic knees with stress fractures. J Orthop Surg (Hong Kong).

[19] Schmidt R, Komistek RD, Blaha JD, Penenberg BL, Maloney WJ (2003). Fluoroscopic analyses of cruciate-retaining and medial pivot knee implants. Clin Orthop Relat Res.

[20] Shakespeare D, Kinzel V, Ledger M (2005). Achieving ligament stability and correct rotational alignment of the femur in knee arthroplasty: a study using the medial pivot knee. Knee.

